# Supplementary material for: Psychological capital and mental health problems among the unemployed in the post-COVID-19 era: Self- esteem as a moderator
Source: PLoS One. 2025 Mar 25;20(3):e0319555. doi: 10.1371/journal.pone.0319555 (PMC11936243; doi:10.1371/journal.pone.0319555)
Supplement: Attachment — Submitted filename: Response comments reviewers_Final.docx [file pone.0319555.s002.docx]

**RE: Detailed responses for reviewers. Manuscript number PONE-D-24-49710**

We would like to express our deep gratitude to the editor and the three reviewers for their thorough evaluation and constructive comments regarding our manuscript. Thanks to your comments, the article was improved significantly.

We are sending herewith our revised manuscript with some revision according to the comments of the three reviewers. Regarding the details, we have addressed the reviewers’ comments as follows:

**REVIEWER #1:**

1. The introduction effectively sets the stage for the importance of the study, linking the COVID-19 pandemic to unemployment and subsequent mental health challenges. This context is highly relevant, and the mention of specific data. helps establish the significance of the issue. However, there is a slight imbalance between global events (bankruptcies, rising inflation) and the focus on the local context. A stronger emphasis on the unique impact in Vietnam would better align the introduction with the focus of the study.

**Author’s response:** We have revised the manuscript as follows:

Line 44 – 50 (Old version)

As a result of this crisis, companies are encountering numerous new challenges, forcing them to implement mass layoffs or reduce their workforce to maintain operating costs [3, 4]. In 2023, more than 260,000 employees from over 1,100 technology companies experienced layoffs, an increase of nearly 58% compared with 2022 [5]. In Vietnam, the wave of layoffs started in early 2023 in diverse fields such as manufacturing, finance, real estate, and technology [6, 7]. Notably, the Ministry of Labor reported that 280,000 Vietnamese workers lost their jobs in the first 6 months of the year [8].

Revised:

As a result of this crisis, companies are encountering numerous new challenges, forcing them to implement mass layoffs or reduce their workforce to maintain operating costs [3, 4]. In 2023, more than 260,000 employees from over 1,100 technology companies experienced layoffs, an increase of nearly 58% compared with 2022 [5]. **Although the global economy has been affected by the COVID-19 pandemic, Vietnam continues to be one of the few developing countries attracting strong Foreign Direct Investment (FDI) flows in the world [6]. However, over-reliance on FDI and exports has made the country's economic performance very vulnerable to external shocks [7]. This has led multinational corporations with branches in Vietnam to drastically cut headcount. Specifically**, in Vietnam, the wave of layoffs started in early 2023 in diverse fields such as manufacturing, finance, real estate, and technology [8, 9]. Notably, the Ministry of Labor reported that 280,000 Vietnamese workers lost their jobs in the first 6 months of the year 2023 [10].

Newly added reference:

[6] Bui TT, Dang LH, Nguyen TMH, Nguyen GTH. Vietnam 2035 Report and Reform of Economic Institutions in 2016-2020. In: World Bank Group [Internet]. Jun 2021 [cited 1 Jan 2024]. Available: <https://documents1.worldbank.org/curated/en/099111106102248474/pdf/P1647370c434f001b08ac408dd5dc320fbc.pdf>

[7] Hiep LH. Vietnam’s Over-reliance on Exports and FDI. 2020 [cited 1 Jan 2024]. Available: <https://www.iseas.edu.sg/wp-content/uploads/2020/09/ISEAS_Perspective_2020_96.pdf>

2. While the introduction outlines the concept of PsyCap, further clarification on how it works as a buffer in the context of job loss would improve understanding. A sentence or two on how each component of PsyCap contributes to mitigating mental health issues would be helpful.

**Author’s response:** We have revised the manuscript as follows:

Line 62-70 (Old version)

Psychological capital (PsyCap) is considered a personal resource that is frequently observed among employees in various business environments [15, 16]. PsyCap is defined as “a state of positive psychological development of an individual”, and characterized by four components: hope, resilience, optimism, and self-efficacy [15]. Based on positive psychology constructs and empirical research, PsyCap refers to an individual’s ability to manage and react to difficult situations, facilitating the development of healthy individuals [15]. In other words, PsyCap, as highlighted by Luthans and Youssef-Morgan [15], is instrumental in preventing mental health issues by fostering adaptive coping strategies and empowering individuals to confront life's challenges with resilience and optimism.

Revised

Psychological capital (PsyCap) is considered a personal resource that is frequently observed among employees in various business environments [17, 18]. PsyCap is defined as “a state of positive psychological development of an individual”, and characterized by four components: hope, resilience, optimism, and self-efficacy [17]. Based on positive psychology constructs and empirical research, PsyCap refers to an individual’s ability to manage and react to difficult situations, facilitating the development of healthy individuals [17]. **Each component of PsyCap plays a vital role in reducing mental health problems. As a synergistic effect, hope and optimism work together create a positive mindset, while self-efficacy and resilience strengthen an individual's belief in their ability to overcome hindrances [17, 19].** In other words, PsyCap, as highlighted by Luthans and Youssef-Morgan [17], is instrumental in preventing mental health issues by fostering adaptive coping strategies and empowering individuals to confront life's challenges with resilience and optimism.

Newly added reference:

[19] Newman A, Ucbasaran D, Zhu F, Hirst G. Psychological capital: A review and synthesis. J Organ Behav. 2014;35: S120–S138. doi: 10.1002/job.1916.

3. Some sentences have unnecessary complexity that could be simplified for clarity and to avoid redundancy. For example line 44, the phrase "encountering numerous new challenges" is a bit redundant.

**Author’s response:** We have revised the manuscript as follows:

Line 44-46 (Old version)

As a result of this crisis, companies are encountering numerous new challenges, forcing them to implement mass layoffs or reduce their workforce to maintain operating costs [3, 4]

Revised:

**As a result of this crisis, companies are forcing them to implement mass layoffs or reduce their workforce to maintain operating costs** [3, 4].

Line 95-98 (Old version)

High self-esteem could also serve as a protective factor against the mental health effects of job loss because people with higher self-esteem engage in more intensive job-search efforts and possess a greater ability to emotionally distance themselves from the distress of involuntary job loss [26].

Revised

**High self-esteem could serve as a protective factor against the mental effects of job loss. People with higher self-esteem tend to engage in more intensive job-search efforts and are better able to emotionally distance themselves from the distress of involuntary job loss [26]**

Line 264 – 267 (Old version):

Additionally, Chen and Lim [43] also suggested that unemployed individuals with high PsyCap tend to have faith in their skills and abilities as well as hold a positive view of future outcomes, thus showing more perseverance in their job-search instead of falling into despair
Revised:

**Additionally, Chen and Lim [43] suggested that unemployed individuals with high PsyCap tend to have faith in their skills and abilities. This faith enables them to maintain a positive view of future outcomes and demonstrate greater perseverance in their job search, rather than succumbing to despair.**

4. For methods section, start with the study design, then describe participants, procedures, and finally, the instruments in a more organized and logical sequence.

**Author’s response:** We have revised the manuscript as follows:

Revised:

**Study design**

This study followed the quantitative method and a cross-sectional design

**Participants**

Participants were laid-off office employees at companies across a variety of industries, including technology, finance, consumer services, and infrastructure services.

**Procedures**

The researcher used an online questionnaire created by Google Survey posted on Facebook and LinkedIn platforms from May 25, 2023 to August 30, 2023, along with a letter calling for sharing to laid-off individuals to participate to receive gifts. The letter of questionnaire fully explained the research objectives, ethics and instructions to the respondents. In addition, to ensure high response rate and reliability, the researcher carefully selected community support groups for laid-off people to deliver the questionnaire. Face-to-face questionnaires were sent to participants through human resources departments of companies in a variety of different fields.

5. line 115, the phrase "office employees" could be more specific. Are these workers from specific companies, or is the sample general across industries?

**Author’s response:** We have revised the manuscript as follows:

Line 115 (Old version): Participants were office employees in the fields of technology, finance, consumer services, and infrastructure services who have been laid off recently.

Revised:

**Participants were laid-off office employees at companies across a variety of industries, including technology, finance, consumer services, and infrastructure services**.

6. The study mentions using "Google Survey" and "social networking platforms such as LinkedIn and Facebook" to collect data. The inclusion of social media platforms can be a good strategy, but the specific method of recruitment (e.g., how the survey link was distributed via these platforms, how the participants were reached) should be further explained.

**Author’s response:** This point was addressed in Reviewer 2’s comment 4.

7. The use of the Rosenberg Self-Esteem Scale (RSES) is appropriate. However, the section could provide more context on how the Vietnamese version was adapted or validated for the population under study. Mention if the scale has been adapted or modified for the specific cultural context or population, and provide additional validation details if possible.

**Author’s response:** We have revised the manuscript as follows:

Revised

**The Rosenberg Self-Esteem Scale was administered to Vietnamese secondary school students, demonstrating excellent reliability and validity [34].**

Reference:

[34] Nguyen DT, Wright EP, Dedding C, Pham TT, Bunders J. Low Self-Esteem and Its Association With Anxiety, Depression, and Suicidal Ideation in Vietnamese Secondary School Students: A Cross-Sectional Study. Front Psychiatry. 2019;10. doi: [10.3389/fpsyt.2019.00698](https://doi.org/10.3389/fpsyt.2019.00698).

8. Some sentences are long and may confuse the reader. Breaking these down into simpler structures would improve readability. For example, the sentence in line 268: "Additionally, Chen and Lim [43] also suggested that unemployed individuals with high PsyCap tend to have faith in their skills and abilities as well as hold a positive view of future outcomes, thus showing more perseverance in their job-search instead of falling into despair."

**Author’s response:** This concern was addressed in Reviewer 1’s 3^rd^ comment.

9. While the discussion refers to behavioral plasticity theory and previous studies, the theoretical implications could be tied more strongly to the observed results, linking the moderation effects of self-esteem more explicitly to the theoretical framework.

**Author’s response:** We have revised the manuscript as follows:

Line 268-282 (Old version)

H2, which states that self-esteem plays a moderating role in the relation between PsyCap and mental health issues, was partially supported by the current findings. Moderation analysis esults showed that self-esteem significantly moderated the relation between PsyCap and stress, depression, and suicidal ideation but not the one between PsyCap and anxiety. Simply put, a high level of self-esteem may act as a buffer between lower PsyCap and depression, stress, and suicidal ideation. These results were consistent with other reports on the moderating role of self-esteem in predicting mental health issues [27, 44, 45]. Our findings were also consistent with behavioral plasticity theory [46], which states that high-self-esteem individuals are less susceptible to environmental events (i.e., being unemployed) than low-self-esteem individuals. The plasticity hypothesis also proposes that high-self-esteem individuals are less affected by organizational events because they tend not to regard social cues and environmental stimuli as guides for their behavior like those with low self-esteem; thus, they are less likely to develop psychological symptoms in response to these environmental stressors. This theory is also supported by Mäkikangas and Kinnunen [47], who showed that self-esteem significantly moderated work stressors and well-being among employees.

Revised:

H2, which states that self-esteem plays a moderating role in the relation between PsyCap and mental health issues, was partially supported by the current findings. Moderation analysis results showed that self-esteem significantly moderated the relation between PsyCap and stress, depression, and suicidal ideation but not the one between PsyCap and anxiety. Simply put, a high level of self-esteem may act as a buffer between lower PsyCap and depression, stress, and suicidal ideation. These results were consistent with other reports on the moderating role of self-esteem in predicting mental health issues [30, 50, 51]. **These results align closely with behavioral plasticity theory [52], which posits that self-esteem can moderate the relationship between environmental stressors (e.g., being unemployed) and adaptive behaviors. In other words, individuals with low self-esteem are more malleable due to external influences, while those with high self-esteem show more stable responses. The theory also proposes that self-esteem plays a buffering role by making individuals with high self-esteem less affected by stressors, as they are less influenced by environmental cues and have greater self-confidence, which enhances their ability to cope with challenges [53]. In the context of our study, it is possible that individuals with high self-esteem are less influenced by stressors related to unemployment, such as the negative emotional impact of job loss, because they rely more on their own self-worth rather than external validation. Additionally, their greater self-confidence and coping abilities may help them maintain psychological stability and resilience in the face of the challenges associated with unemployment. Our results also extend prior findings supporting behavioral plasticity theory, such as those reported by Mäkikangas and Kinnunen [54]**, who showed that self-esteem significantly moderated work stressors and well-being among employees.

New add reference:

[53] Saks AM, Ashforth BE. The role of dispositions, entry stressors, and behavioral plasticity theory in predicting newcomers’ adjustment to work. J Organ Behav. 2000;21: 43–62. doi: 10.1002/(sici)1099-1379(200002)21:1%3C43::aid-job985%3E3.0.co;2-w.

10. The limitations section could be more specific. For example, addressing the self-report bias could be enhanced with a more detailed discussion on how future studies might mitigate such bias (e.g., through longitudinal studies or multi-source data collection).

**Author’s response:** We have revised the manuscript as follows:

Line 312-319 (Old version)

This study has several limitations that must be addressed. First, it implemented a cross sectional study design, which makes it difficult to derive causal relations between study variables. Second, the study variables were measured using self-report instruments, possibly causing response bias and affecting the results. Finally, we only collected data from unemployed individuals with permanent employment. Bjarnason and Sigurdardottir [57] suggested that employees under temporary employment suffer from more severe psychological distress than those under permanent employment. Hence, future studies could offer a deeper understanding of types of employment by comparing mental well-being between unemployed individuals with temporary employment and those with permanent employment

**Revised:**

This study has several limitations that should be addressed in subsequent studies. First, the cross-sectional design limits the ability to draw causal conclusions between the study variables. Longitudinal studies would be more effective in establishing causal relationships and tracking changes over time. Second, the reliance on self-report instruments introduces the possibility of response bias, which may affect the validity of the results. This limitation could be mitigated in future investigations by incorporating multi-source data collection, such as using both self-report and observational measures, or adopting longitudinal designs that capture psychological outcomes over time. Additionally, the study used convenience sampling via LinkedIn and Facebook, which may introduce selection bias, as individuals who use these platforms might not represent the broader population, particularly in terms of socio-demographic factors or mental well-being. Therefore, subsequent studies should consider more diverse sampling methods to improve generalizability. Finally, the sample was limited to unemployed individuals seeking permanent employment, while previous studies have shown that individuals in temporary employment often experience higher psychological distress than those in permanent positions [57]. Further investigations could explore this distinction by comparing the mental well-being of unemployed individuals with temporary employment to those with permanent employment, offering a more comprehensive understanding of employment types and their effects on mental health.

**REVIEWER #2:**

The article can be accepted for publication. There are, however some issues that need to be clarified:
1. As for the clinical implications, the authors suggested PCI and self-esteem enhancement. Should these interventions be given to employees after a layoff, or should these interventions be included in the employee mental health programs while the employee is still employed?

**Author’s response:** We have revised the manuscript as follows:

Line 297-309 (Old version)

Our findings indicate that low PsyCap is a significant risk factor for mental health problems among the unemployed. Thus, enhancing one’s PsyCap should be a main focus in improving their mental health during an economic crisis. This may be done through psychological capital intervention (PCI), a novel intervention program developed by Luthans et al. [50] and expanded upon by Dello Russo and Stoykova [51]. PCI is a microintervention that typically consists of a one- to four-hour group session designed to adopt various methods to improve all four PsyCap components among participants (i.e., efficacy, hope, resilience, and optimism) [50]. Additionally, PCI has shown effectiveness in improving mental well-being [52] and reducing depression [53]. These findings also suggest that interventions for unemployed people incorporate self-esteem enhancement to foster a greater sense of mental well-being after a layoff. Indeed, interventions that include self-esteem enhancement have been suggested to be effective in reducing suicidal ideation [54]. To improve self-esteem, cognitive behavior therapy and compassion focused therapy have demonstrated efficacy in treating individuals with low self-esteem [55, 56].

Revised:

Our findings indicate that low PsyCap is a significant risk factor for mental health problems among the unemployed. Thus, enhancing one’s PsyCap should be a main focus in improving their mental health during an economic crisis. This may be done through psychological capital intervention (PCI), a novel intervention program developed by Luthans et al. [57] and expanded upon by Dello Russo and Stoykova [58]. PCI is a microintervention that typically consists of a one- to four-hour group session designed to adopt various methods to improve all four PsyCap components among participants (i.e., efficacy, hope, resilience, and optimism) [57]. PCI has shown effectiveness in improving mental well-being [59] and reducing depression [60]. Accordingly, applying this program to unemployed individuals may also provide significant benefits in addressing mental health challenges during such crises. **Additionally, previous intervention studies have demonstrated that PCI can reduce depression and improve job performance in employees even at follow-up periods, highlighting its sustained benefits [60, 61]. Given these findings, these interventions could also be integrated into employee mental health programs while proactively**.

These findings also suggest that interventions for unemployed people incorporate self-esteem enhancement to foster a greater sense of mental well-being after a layoff. Indeed, interventions that include self-esteem enhancement have been suggested to be effective in reducing suicidal ideation [62]. To improve self-esteem, cognitive behavior therapy and compassion focused therapy have demonstrated efficacy in treating individuals with low self-esteem [63, 64].

References:

[62] Song R, Sun N, Song X. The Efficacy of Psychological Capital Intervention (PCI) for Depression From the Perspective of Positive Psychology: A Pilot Study. Front psychol. 2019;10. doi: 10.3389/fpsyg.2019.01816

[61] Luthans F, Avey JB, Avolio BJ, Peterson SJ. The development and resulting performance impact of positive psychological capital. Hum Resour Dev Q. 2010;21: 41–67. doi: 10.1002/hrdq.20034.

2. Lines 328-330, what socio-economic development are you referring to here?

**Author’s response:** We have revised the manuscript as follows:

Line 328-330 (Old version)

Considering this, companies and governmental organizations ought to implement policies that offer psychological support to jobless workers to ensure socioeconomic development.

- Revised to: Considering this, companies and governmental organizations ought to implement policies that offer psychological support to jobless workers to promote **economic growth, reduce social welfare burdens, and enhance social stability.**

3. Lines 330-331, I wonder where in the paper was this explained to include this in the conclusion (especially on building resilience)? You should rephrase this to capture the essence of your whole paper.

**Author’s response:** We have revised the manuscript as follows:

Line 330-331 (Old version)

Finally, employees should understand that in order to build resilience in the post COVID-19 era, they must enhance their PsyCap and self-esteem.

Revised:

Finally, employees can mitigate mental distress during challenging events such as unemployment, particularly in the post-COVID-19 era, by enhancing their psychological capital (PsyCap) and self-esteem.

**REVIEWER #3:**

Specific Comments and Recommendations:
1 Introduction and Objectives
1.1. Rephrase lines 104–106 to better align with the objectives of the study.

**Author’s response:** We have revised the manuscript as follows:

Line 104-106 (Old version)

Drawing from the literature, this study aims to investigate the level of depression, anxiety, and stress among employees experiencing job loss. It also seeks to examine the buffering effects of PsyCap and self-esteem in the relation between job loss and mental health issues.

Revised line 105 - 106

**Drawing from the literature, this study aims to investigate the level of depression, anxiety, and stress among employees experiencing job loss. The study also sought to examine the buffering effect of PsyCap on mental health problems, and the role of self-esteem in the relation between PsyCap and mental health problems of laid-off employees in Vietnam.**

2. Methods
2.1. Consider organizing the Methods section into distinct subsections, including:
♣ Study design
♣ Study population
♣ Inclusion/exclusion criteria
♣ Measures (e.g., primary and secondary outcomes, exposure variables, confounders, and effect modifiers)
♣ Study procedures
♣ Sample size considerations
♣ Data analysis

**Author’s comment:** This point was addressed in reviewer 1'st 4^th^ comment

In the Measures subsection:
2.2. Provide details on how each variable included in the final analysis was treated (e.g., cut-off values used).

**Author’s respond:** This point was addressed in Reviewer 3’s 2.3. Comment (the next comment)

2.3. Include information on the minimum and maximum scores expected for each of the tools used to assess the outcome variables, giving readers more context.

**Author’s response:** We have revised the manuscript as follows:

Revised

**Psychological Capital**

This was measured using the 12-item Psychological Capital Questionnaire - Short form (PCQ) [32], which was standardized in Vietnamese by Ha and Trung [33]. It consists of four subscales: hope (4 items), resilience (3), optimism (2), and self-efficacy (3). All items are rated on a six-point Likert scale from 1 (strongly disagree) to 6 (strongly agree). Each of the four PCQ subscale scores is calculated by taking the mean (average) of all items in the scale. **The overall PsyCap score is then calculated by taking the mean of all PCQ items ranging from 12 (Lowest score) to 72 (Highest score).** The overall PsyCap score is then calculated by taking the mean of all PCQ items. The internal consistency of the scale was 0.92.

**Self-esteem**

This was measured using the 10-item Rosenberg Self-Esteem Scale (RSES) [27]. All items are rated on a four-point Likert scale from 1 (strongly disagree) to 4 (strongly agree). A total score of 10 to 40 is calculated by summing the scores of the ten items, with a higher total score indicates a higher sense of self-esteem. In which, scoring for items 2, 5, 6, 8, and 9 will be reversed. **The Rosenberg Self-Esteem Scale was administered to Vietnamese secondary school students, demonstrating excellent reliability and validity [34].** The internal consistency of the scale was 0.9.

**Mental health problems**

These were measured using the 21-item Depression Anxiety Stress Scales - Short Form (DASS-21) [35], which has been standardized in Vietnamese by Le et al. [36]. It is composed of three subscales with seven items each: depression (DE), anxiety (AN), and stress (ST). All items are rated on a three-point Likert scale from 0 (Did not apply to me at all) to 3 (Applied to me very much or most of the time). Total scores are multiplied by 2 to reflect the original 42-item scale. **Higher scores indicate greater levels of depression, anxiety, and stress. The scores range from 0 to 21 for each subscale and from 0 to 63 for the total scale. The DASS-21 provides standardized cut-off scores to categorize the severity of symptoms for each subscale. For depression: normal (0–9), mild (10–13), moderate (14–20), severe (21–27), and extremely severe (28 or higher). For anxiety: normal (0–7), mild (8–9), moderate (10–14), severe (15–19), and extremely severe (20 or higher). For stress: normal (0–14), mild (15–18), moderate (19–25), severe (26–33), and extremely severe (34 or higher).** The internal consistencies of the three subscales were 0.76 (depression), 079 (anxiety), and 0.91 (stress).

**Suicidal ideation**:

This was measured using item 9 of the Patient Health Questionnaire-9 (PHQ-9) [37], which states “Thoughts that you would be better off dead or of hurting yourself in some way.” This has been standardized in Vietnamese by Nguyen et al. [38]. This measure was selected because the item has demonstrated a high degree of reliability in predicting suicidal ideation across different community contexts [39, 40]. This item is rated on a three-point Likert scale from 0 (not at all) to 3 (nearly every day). Higher scores indicate high levels of suicidal ideation. The reliability found in this study was 0.89.

**Suicidal is measured in 1 sentence in PHQ-9 so no need to revise**

2.4. Address sample size considerations: While the sample size may be adequate for the study’s main objective, include details on how the sample size calculation was performed.

**Author’s response:** We have revised the manuscript as follows:

Revised:

Sample size

**The sample size was calculated by G-Power software (version 3.1.9.7) based on a power of 0.95, an alpha of 0.05, and an effect size of 0.15 for linear multiple regression. The necessary total sample size for the current study was 166.**

2.5. Provide information on how missing or implausible values were handled in the analysis.

**Author’s response:** We have revised the manuscript as follows:

**Revised**

**For each study sample, we imputed missing data using the predictive mean matching (PMM) method [41, 42]. Analysis showed that the current study had no missing data.**

Reference:

[41 Van Buuren S. Flexible imputation of missing data. Boca Raton, Fl: Crc Press, Taylor & Francis Group; 2018.

[42] Austin PC, White IR, Lee DS, van Buuren S. Missing data in clinical research: a tutorial on multiple imputation. Can J Cardiol. 2021;37(9):1322–31.

2.6. Specify whether the distribution of the variables was checked prior to analysis.

**Author’s response:** We have revised the manuscript as follows:

Line 178 – 181

Revised:

Variables were checked for distribution before analysis, skewness values between -1 and +1 and kurtosis values between -2 and +2 are typically considered acceptable for normality. Values outside of these ranges suggest deviations from a normal distribution.

3. Data Analysis
3.1. Specify the type of regression analysis performed.

**Author’s response:** We have revised the manuscript as follows:

Revised

**Finally, we performed hierarchical regression analysis and used the SPSS PROCESS macro, Model 1 (version 3.0)**

3.2. Clearly explain why PROCESS Model 1 was chosen for moderation.

**Author’s response:** We have revised the manuscript as follows:

**PROCESS Model 1 was chosen because it is specifically designed to estimate moderation effects when a single moderator is present, aligning with the requirements of our hypothesiss [43].**

New Reference:

[43] Hayes AF. Introduction to Mediation, Moderation, and Conditional Process Analysis: A Regression-Based Approach. 3rd ed. Guilford Publications; 2017.

3.3. Justify the decision to control demographic variables in all models.

**Author’s response:** We have revised the manuscript as follows:

Line 228 – 231 (Old version)

Revised:

Control variables are important in a moderator model because they support account for extraneous factors that might influence the dependent variable, ensuring a more accurate estimation of the relationships among the key variables of interest [43]. Therefore, we use demographic factors as control variables for all moderator models.

New References

[43] Hayes AF. Introduction to Mediation, Moderation, and Conditional Process Analysis: A Regression-Based Approach. 3rd ed. Guilford Publications; 2017.

4. Tables and Figures
4.1. Revise table and figure titles to explicitly reflect "what," "when," and "where," ensuring they align with the data presented.

**Author’s response:** We have revised the manuscript as follows:

**Revised Name Table:**

**Table 1: Sociodemographic characteristics of post-COVID unemployment study in Vietnam**

**Table 2: Levels of stress, anxiety, and depression among post- COVID unemployed people in Vietnam**

**Table 3: Correlations, means, and standard deviations among variables**

4.2. For Table 1, describe the 3rd to 6th columns in the text.

**Author’s response:** We have revised the manuscript as follows:

Revised:

When comparing mean scores based on demographic variables, no significant differences in mental health problems were observed across gender and age groups. However, among marital status and education groups, the mean scores for mental health problems (depression, anxiety, stress, and suicidal ideation) were higher in the postgraduate education and divorced categories compared to other groups.

4.3. Include 95% CI for regression results, and organize the table to clearly distinguish between crude and adjusted results.

**Author’s respond:** We have revised the table 4 as suggested with crude results and 95% CI added.

Due to adjustment of some tables, we have revised the reporting of decimal places throughout the manuscript, changing it from three decimal places to two decimal places.

4.4. For very small p-values, avoid reporting them as 0.000; instead, use p < 0.01.

**Author’s respond:** We have revised the p-value in the Tables.

4.5. Ensure information presented in the Results section is reflected in the tables/figures.

**Author’s respond:** We have ensured that all key findings mentioned in the Results section are clearly summarized in the existing tables/figures, with corresponding details fully described in the text.

5.. Result
5.1. Expand the text on the regression analysis results to include the magnitude of associations, providing a more comprehensive view of their strength and relevance.

**Author’s response:** We have revised the manuscript as follows:

Revised

**Line 245 - 248**

When stress is entered as the outcome, Table 4 showed that PsyCap (β = ˗.37, p < 0.01) and self-esteem negatively predicted stress (β = ˗.24, p < 0.01). **Specifically, these two variables were found to explain 38% of variance in level of stress (*R*^2^ = .38, p < 0.01)**. The interaction (PsyCap × self-esteem) also negatively predicted stress (β = ˗.10, p < 0.05).

**Line 294 - 297**

Table 4 showed that PsyCap negatively predicted anxiety (β = ˗.33, p < 0.01). Self-esteem also negatively predicted anxiety (β = ˗.24, p < 0.01). **PsyCap and self-esteem were found to explain 36% of variance in level of anxiety (*R*^2^ = .36, p < 0.01).** The interaction (PsyCap × self-esteem) did not negatively predict anxiety (p > 0.05)

**Line 300 – 305**

The results (Table 4) showed that PsyCap negatively predicted depression (β = ˗.31, p < 0.01). Self-esteem negatively predicted depression as well (β = ˗.41, p < 0.01**). PsyCap and self-esteem were found to explain 57% of variance in level of depression (*R*^2^ = .57, p < 0.01).** The interaction (PsyCap × self-esteem) also negatively predicted depression (β = ˗.10, p < 0.05). Overall, these results suggest that self-esteem can moderate the relation between PsyCap and depression among unemployed people.

**Line 314 - 319**

The results (Table 4) showed that PsyCap negatively predicted suicidal ideation (β =  ˗.25, p < 0.01). Self-esteem did not predict suicidal ideation (p > 0.05). **PsyCap and self-esteem were found to explain 15% of the variance in suicidal ideation (R² = .154, p < .01).** However, the interaction (PsyCap × self-esteem) positively predicted suicidal ideation (β = .01, p < 0.05). Overall, these results suggest that low self-esteem can moderate the relation between PsyCap and depression among unemployed people

5.3. Double-check figures mentioned on lines 213 and 218.

**Author’s respond:** Thank you for your comment. However, the moderating effect was not significant for Anxiety so there was no figure for the Anxiety variable (line 213). As a result, the Figure for Depression is numbered as Fig 2. (Line 218).

5.4. Correct the term "independent" on line 191 to "dependent."

**Author’s response:** We have revised the manuscript as follows:

Line 190 (Old version): **Model 1: Stress as an independent variable**

Revised:

Line 243: **Model 1: Stress as a dependent variable**

Line 279: **Model 2: Anxiety as a dependent variable**

Line 285: **Model 3: Depression as a dependent variable**

Line 299; **Model 4: Suicidal ideation as a dependent variable**

6. Discussion and Limitations
6.1. Acknowledge the potential for selection bias due to convenience sampling via LinkedIn and Facebook in the Discussion section.

**Author’s respond:** This comment has been addressed in Reviewer 1’s comment 10^th^.

7. General Recommendations
7.1. Improve the interpretation of results by emphasizing their implications while maintaining clarity.

**Author’s respond:** This comment has been addressed in Reviewer 1’s comment 9^th^ and Reviewer 2’s comment 1^st^.

7.2. Ensure consistent formatting and adherence to reporting guidelines throughout the manuscript.

**Author’s respond:** We have carefully reviewed the entire manuscript to ensure compliance with the relevant reporting standards and to maintain consistent formatting throughout.

o Although ethics approval is mentioned, explicitly state how consent was obtained (e.g., checkbox or written agreement in the online form).

A**uthor respond:** We have addressed this concern in Reviewer 1’s comment 4^th^.
